# Subunits of adaptor protein complex-1 make distinct contributions to the virulence of *Cryptococcus neoformans*

**DOI:** 10.1128/iai.00100-26

**Published:** 2026-08-04

**Authors:** Kabir Bhalla, Eddy Sánchez-León, Yu-Hsuan Huang, Guanggan Hu, Anisha Neogi, Alba Torres-Cano, Oscar Zaragoza, Pauline Johnson, James W. Kronstad

**Affiliations:** 1 Michael Smith Laboratories, University of British Columbia 8166, Vancouver, BC, Canada; 2 Department of Microbiology and Immunology, University of British Columbia 198130, Vancouver, BC, Canada; 3 Department of Oral Biological and Medical Sciences, University of British Columbia 8166, Vancouver, BC, Canada; 4 Mycology Reference Laboratory, National Centre for Microbiology, Instituto de Salud Carlos III 91837, Majadahonda, Madrid, Spain; 5 Center for Biomedical Research in Network in Infectious Diseases (CIBERINFEC-CB21/13/00105), Instituto de Salud Carlos III 38176, Madrid, Spain; University of Illinois Chicago, Chicago, Illinois, USA

**Keywords:** endomembrane trafficking, phagocytosis, fungal pathogenesis, cytokines, granuloma

## Abstract

Cryptococcal meningoencephalitis is among the most prevalent invasive fungal diseases, posing a threat to immunocompromised individuals and representing a growing global health concern. The mechanisms of cryptococcal trafficking of virulence factors during disease are incompletely understood. Adaptor protein (AP) complexes play crucial roles in intracellular trafficking by orchestrating the sorting of macromolecular cargo and serving as essential components of the endocytic and secretory pathways. In a recent study, we demonstrated that *Cryptococcus neoformans* cells lacking the AP-1 complex subunits exhibit impaired elaboration of virulence factors and fail to survive in the harsh conditions of the macrophage phagolysosome. Although we characterized the phenotypes of AP-1 deficient cells *in vitro*, the contribution of this complex to pathogenesis remains unexplored. In this study, we show that mutants deficient in AP-1 complex subunits are either avirulent or exhibit attenuated virulence in a murine inhalational model. Loss of the small subunit resulted in the formation of granuloma-like lesions in mouse lungs with early containment of infection but eventual mortality, whereas mutants lacking the large subunits were rapidly cleared by mice. The delayed onset of disease in mice caused by mutants lacking the small subunit was marked by delayed weight loss and increased respiration rate, yet the mutant exhibited enhanced dissemination during late-stage infection, coinciding with waning immune responses and elevated collagen deposition. These findings demonstrate that deficiencies in the AP-1 complex impair *C. neoformans* virulence, reveal distinct roles for individual subunits, and identify the complex as a potential target for therapeutic intervention in cryptococcosis.

## INTRODUCTION

A network of proteins controls the trafficking and sorting of macromolecular cargoes within post-Golgi compartments of eukaryotic cells (1–3). The adaptor protein (AP) complexes are essential components of the endocytic and secretory trafficking machinery that mediate cargo selection and vesicle formation (2–5). AP complexes are known to associate with the cytosolic face of membranes, where they facilitate the creation of supramolecular assemblies known as protein coats. These complexes recognize and bind to specific sorting motifs in the cytoplasmic tails of transmembrane cargo proteins (6) and recruit clathrin along with accessory factors to initiate vesicle formation (3). Through these interactions, AP complexes concentrate cargo into clathrin-coated vesicles (CCVs) and mediate their delivery from donor membranes to appropriate target organelles (3). The AP complexes are highly conserved across all eukaryotic organisms, where they play essential roles in maintaining normal physiological functions (3, 5).

Post trans-Golgi network (TGN) trafficking is highly complex and involves sorting to various organelles and the plasma membrane (5). Multiple AP complexes participate in these processes. To date, five AP complexes have been identified: AP-1, AP-2, AP-3, AP-4, and AP-5 (2, 3). While fungi possess homologs of all subunits of the AP-1, AP-2, and AP-3 complexes, most species have lost the AP-4 and AP-5 complexes (7). Each AP complex contains four homologous subunits: two large, one medium, and one small subunit (1, 2, 5, 8). Despite the structural similarities, these complexes differ in their subcellular localization and function (2). AP-1 and AP-2 are clathrin-associated complexes that mediate vesicle formation at the TGN and plasma membrane, respectively (5, 9). The AP-3 complex functions in both clathrin-dependent and clathrin-independent pathways and is known to localize to the cytosol, TGN, late endosomes, lysosomes, and lysosome-related organelles (2). In yeast and mammalian cells, AP-3 mediates the sorting of vacuolar and lysosomal membrane proteins (2, 5).

AP-1 is a clathrin-associated heterotetrameric complex localized at the TGN and early endosomes, where it is involved in several distinct sorting pathways, including retrograde transport (3, 6, 10). AP-1 mediates bidirectional transport between the TGN and endosomes and, in polarized cells, also facilitates recycling of cargo from endosomes to the plasma membrane (5, 8, 11). AP-1 contributes to basolateral trafficking in epithelial cells and to the biogenesis and maturation of secretory granules including Weibel-Palade bodies (8, 12, 13). The AP-1 complex subunits also colocalize with newly synthesized cargo and retrieve missorted proteins (5, 13). In the model yeast *Saccharomyces cerevisiae*, disruption of AP-1 does not impact cell growth but impairs the retrieval of late Golgi resident proteins from endosomes, indicating a specific role in retrograde transport (14, 15). However, later studies showed that AP-1 deficiency also results in mislocalization of intracellular proteins to the cell surface (16, 17). In *Arabidopsis*, loss of the AP-1 medium subunit impairs both secretory and vacuolar trafficking, suggesting functions in secretory vesicle formation and plasma membrane transport (18, 19). Collectively, AP-1 functions across eukaryotes at the TGN, endosomes, and plasma membrane to regulate polarized sorting and secretion (14). It is essential for processes, such as basolateral and apical transport, endosomal recycling, and formation of specialized secretory organelles.

The AP-1 complex is also clinically significant because defects have been linked to various human genetic disorders, and the complex is exploited by pathogens, such as HIV to facilitate infection (5, 9). The physiological importance of AP-1 is underscored by the embryonic lethality observed in mice lacking AP-1 subunits (9). AP-1 also plays critical roles in the virulence of many pathogenic and parasitic organisms. In *Giardia lamblia*, AP-1 is involved in the anterograde protein transport to peripheral vacuoles, which are lysosome-like organelles located underneath the plasma membrane (20). In *Leishmania mexicana*, the AP-1 complex was found to contribute to flagellum biogenesis. Additionally, AP-1 mutants were hypersensitive to elevated temperature and perturbations in membrane lipid composition (21).

In this study, we examined the role of the AP-1 complex in the virulence of *Cryptococcus neoformans*, the causative agent of life-threatening fungal meningoencephalitis in immunocompromised individuals (22, 23). In recent work, we demonstrated that loss of subunits of AP-1 impairs elaboration of key virulence factors, including polysaccharide capsule, melanin, and urease, disrupts vacuolar function, and perturbs polyphosphate metabolism in *C. neoformans* (24). Additionally, deletion of individual AP-1 subunits produces distinct pleiotropic phenotypes under stress conditions and ablates survival in macrophages, further supporting a role for this complex in fungal virulence. However, whether loss of these proteins is required for virulence *in vivo* is yet to be described. To extend our previous work and assess the role of the AP-1 complex in pathogenesis, we infected BALB/c mice with *C. neoformans* mutants lacking either the large or small subunits of the complex. We discovered that mutants deficient in AP-1 were either avirulent or showed attenuated virulence in the murine inhalational model. Loss of the small subunit led to pulmonary granuloma formation, early infection control, but eventual mortality, whereas mutants lacking the large subunits were rapidly cleared by the host. Small subunit mutants also exhibited delayed disease onset and enhanced dissemination during late infection, together with a waning immune response in mice. Together, our findings indicate that AP-1 complex deficiencies impair *C. neoformans* virulence, reveal distinct roles for the subunits, and identify the complex as a potential target for control of cryptococcosis.

## RESULTS

### Disruption of AP-1 complex adaptins impairs virulence in murine models

**Fig 1:**
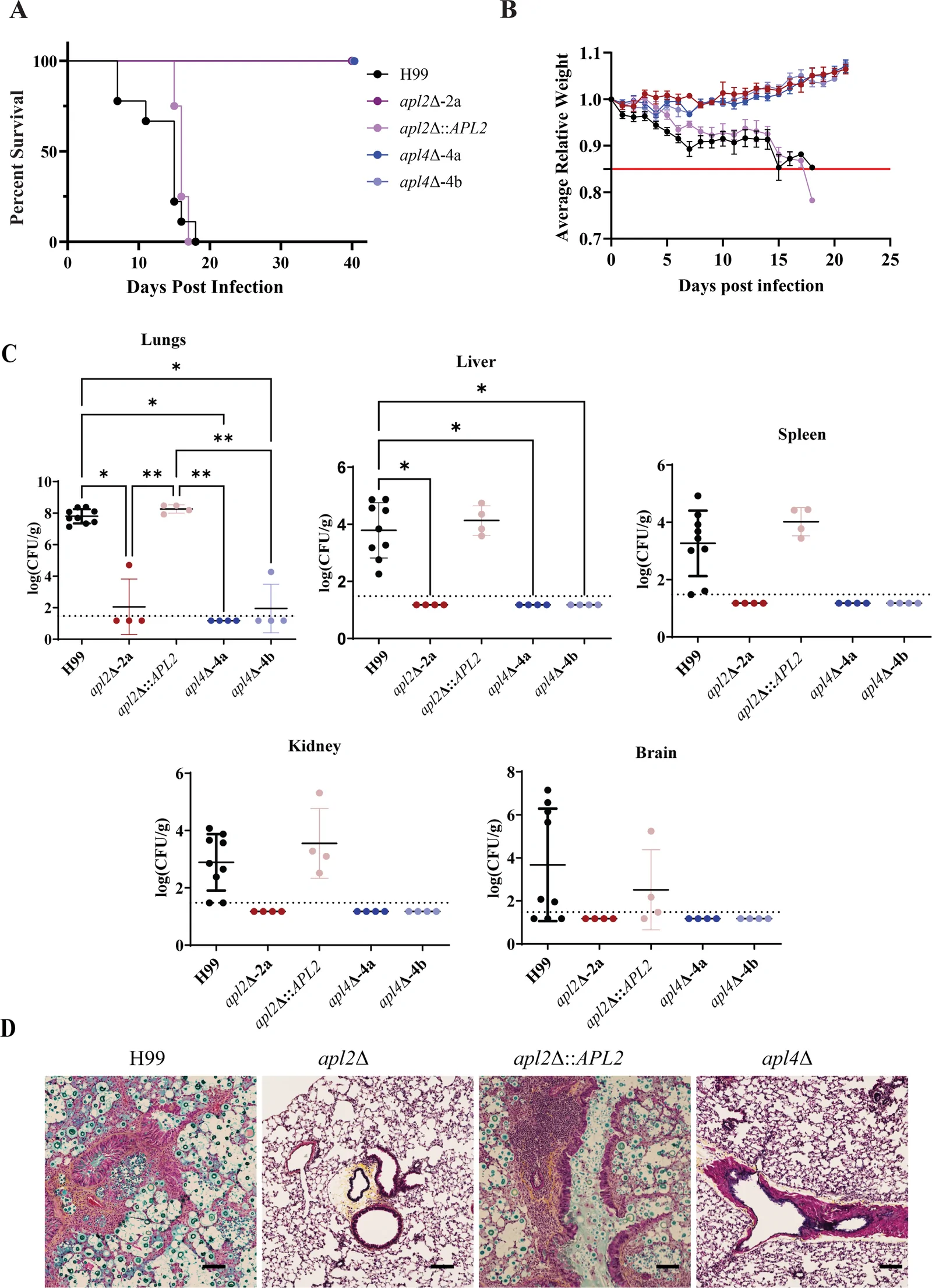
Mutants lacking large subunits of the AP-1 complex are avirulent. (**A**) Survival of BALB/c mice intranasally inoculated with cells of the WT (H99), *apl2*∆, *apl4*∆ (two independent mutants), or the *apl2*∆::*APL2* (complemented) strains. (**B**) The average daily mouse weight, relative to initial weight, was used to track disease progression during the first 20 days post-infection (dpi). A solid red horizontal line indicates the threshold for euthanasia at 85% of the initial weight. Error bars represent the standard deviation (SD) from all surviving mice (*n* = 10 for WT, *n* = 4 for *apl2*∆, *apl4*∆, or *apl2*∆::*APL2* complemented strains). (**C**) The fungal burden for mice infected with each strain was determined by measuring colony-forming units (CFUs). The dotted black line indicates the limit of detection for each organ. (**D**) Representative histopathological micrographs of lung tissue from mice infected with WT, *apl2*∆, *apl4*∆, or *apl2*∆::*APL2* complemented strains, collected at the experimental endpoint and stained with Movat’s Pentachrome. Scale bars = 100 µm. Data are presented as mean ± SD. Significance indicated as *, *P* < 0.05; **, *P* < 0.01; one-way ANOVA.

Loss of the AP-1 complex in *C. neoformans* impairs the elaboration of key virulence factors and renders the fungus hypersensitive to elevated temperatures and phagocytic killing (24). These phenotypes align with findings in pathogenic protozoa, where the AP-1 adaptor complex plays a role in virulence (21, 25). Given the phenotypes of the AP-1 complex mutants in *C. neoformans*, we hypothesized that loss of the complex would also impact virulence in a mouse model of cryptococcosis. To test this idea, we first assessed the ability of the mutants lacking the large subunits known as adaptins (*apl2*∆ and *apl4*∆) to cause disease. We infected BALB/c mice by intranasal inoculation with the wild-type (WT) strain (H99), the mutants (*apl2*∆ or two independent *apl4*∆ mutants), or the *apl2*∆::*APL2* complemented strain and monitored disease progression and survival. Unlike mice infected with the WT or complemented *apl2*Δ::*APL2* strains, which succumbed by days 19 and 18, respectively, those infected with *apl2*Δ or *apl4*Δ mutants showed no disease symptoms or mortality, indicating that the mutants were avirulent (Fig. 1A). We also examined the role of the medium subunit, Apm1*,* in virulence by intranasally inoculating BALB/c mice with two independent *apm1*∆ mutant strains and the complemented *apm1*∆::*APM1* strain. Consistent with phenotypes of the *apl2*∆ and *apl4*∆ mutants, the *apm1*∆ strains were avirulent, and mice infected with these mutants showed no signs of disease or mortality (Fig. S1A). The impaired virulence of the *apl2*∆ and *apl4*∆ mutants was also evident when disease progression was monitored by weight loss for 20 days post-inoculation. Mice infected with the WT and *apl2*∆::*APL2* strains lost weight for 15 days, whereas the mice infected with *apl2*∆ or *apl4*∆ mutants continued to gain weight (Fig. 1B). An analysis of fungal burden in organs collected at the experimental endpoint (for WT and *apl2*∆::*APL2*-infected mice) and at day 40 (predetermined endpoint for *apl2*∆ and *apl4*∆-infected mice) revealed significantly lower colony-forming units (CFUs) in the lungs of mice infected with the mutants, with no detectable CFUs in the liver, spleen, kidneys, or brain (Fig. 1C). A similar pattern was observed for the *apm1*∆ mutants, which also displayed substantially reduced fungal burdens in lungs and spleen (Fig. S1B). Histological analysis of infected lung tissue revealed an abundance of fungal cells in mice infected with the WT or complemented strains, whereas no fungal cells were detected in the lungs of mice infected with the *apl2*∆ or *apl4*∆ mutants (Fig. 1D). Mice infected with the *apm1*∆ mutants showed a similar outcome, with no fungal cells observed in lung tissue (Fig. S1C). Lung sections of mice infected with the WT or complemented strains revealed marked disruption of normal architecture, with extensive immune cell infiltration and mucus-filled airways. On the other hand, lung sections of mice infected with the *apl2*∆ and *apl4*∆ mutants showed normal lung architecture with minimal immune cell infiltration and clear airways (Fig. 1D). Taken together, these findings indicate that the medium and large AP-1 adaptin subunits are required for the ability of *C. neoformans* to cause disease.

### Loss of the AP-1 small subunit results in delayed disease in mice

**Fig 2:**
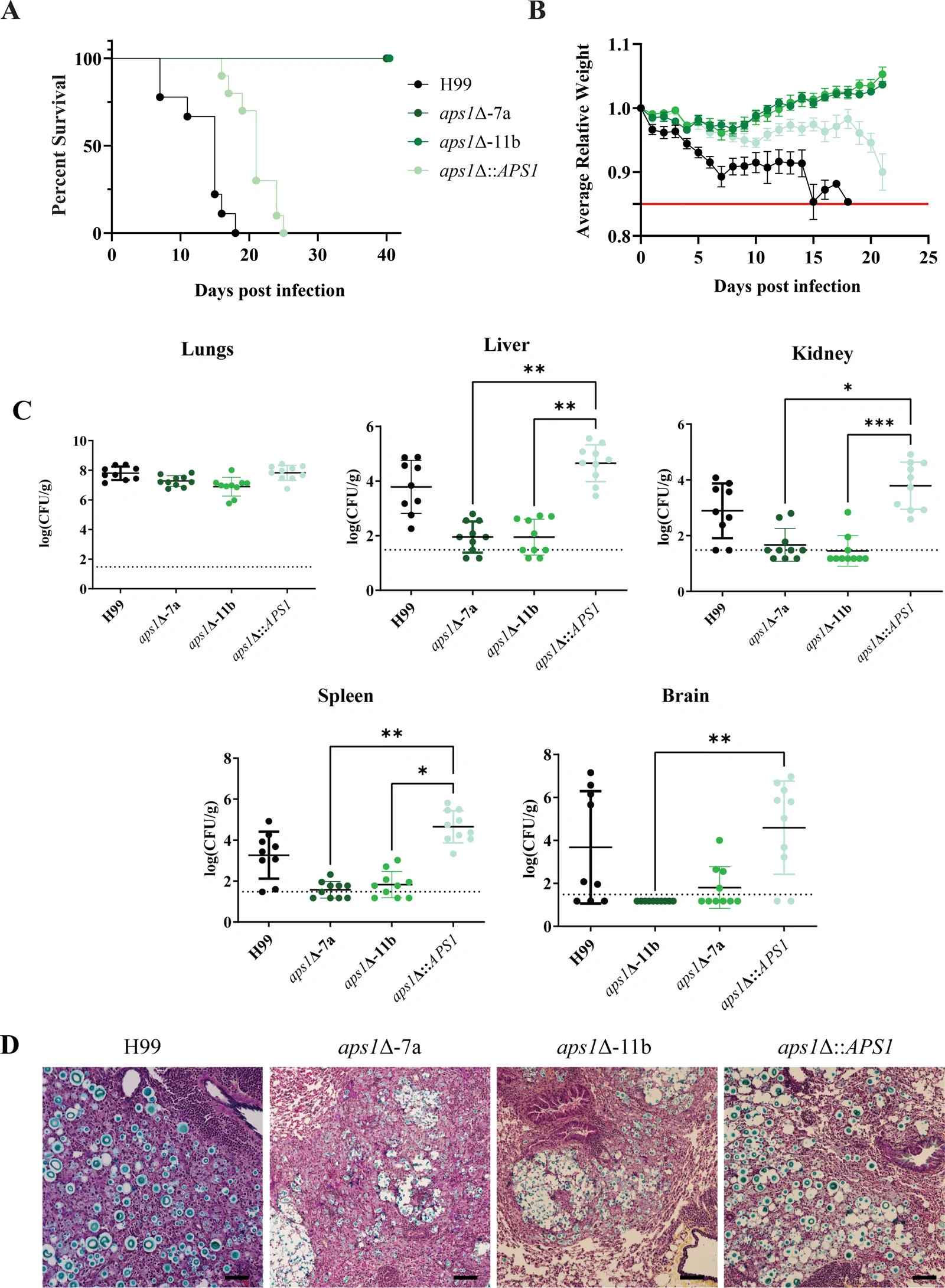
Loss of the AP-1 small subunit produces a prolonged infection, with normal weight maintenance despite WT levels of fungal burden. (**A**) Survival of BALB/C mice intranasally inoculated with cells of the WT strain (H99), two independent *aps1*Δ mutants or *aps1*∆::*APS1* complemented strain. (**B**) The average daily mouse weight, relative to initial weight, was used to track disease progression during the first 20 days post-infection. A solid red horizontal line indicates the threshold for euthanasia at 85% of the initial weight. Error bars represent standard deviation (SD) from all surviving mice (*n* = 10). (**C**) Colony-forming units (CFUs) were measured to determine fungal burden in lungs, liver, kidney, spleen, and brain for mice infected with each strain. The dotted black line indicates the limit of detection for each organ. (**D**) Representative histopathological micrographs of lung tissue from mice infected with WT, *aps1*∆−7a, *aps1*∆−11b, or *aps1*∆::*APS1* complemented strains, collected at experimental endpoint, and stained with Movat’s Pentachrome. Scale bars = 100 µm. Data are presented as mean ± SD. Significance indicated as *, *P* < 0.05; **, *P* < 0.01; ***, *P* < 0.001; one-way ANOVA. The WT control shown in Fig. 1 and 2 is identical, as the experiments were performed concurrently.

*C. neoformans* mutants lacking the small subunit (*aps1*∆) exhibited phenotypes distinct from those deficient in large subunits (*apl2*∆, *apl4*∆), indicating that individual AP-1 subunits potentially perform distinct, nonredundant functions (24). Notably, the *aps1*∆ mutants displayed relatively greater resistance to stress conditions, such as elevated temperature and cell wall stress, compared with *apl2*∆ and *apl4*∆ mutants (24). We therefore hypothesized that *aps1*∆ mutants would exhibit enhanced survival and increased virulence in the host. To test this idea, we challenged BALB/c mice by intranasal inoculation with the WT strain, two independent *aps1*∆ mutants, or the *aps1*∆::*APS1* complemented strain and monitored disease progression and survival. Unlike mice infected with the WT or complemented *aps1*Δ::*APS1* strain, which succumbed by days 19 and 25, respectively, those infected with *aps1*∆ mutants showed no disease symptoms or mortality by day 40 post-infection (Fig. 2A). The difference in virulence for the *aps1*∆ mutants, compared with mutants lacking the medium and large subunits (Fig. 1; Fig. S1), was also evident when disease progression was monitored by weight loss for 20 days post-inoculation. Mice infected with the WT or the *aps1*∆::*APS1* strains lost weight for 15 and 18 days, respectively, whereas the mice infected with *aps1*∆ mutants continued to gain weight and did not show any signs of disease throughout the 40-day experiment (Fig. 2B). Surprisingly, fungal burden analysis of organs collected at the experimental endpoint (for WT and *aps1*∆::*APS1*) and at day 40 (predetermined endpoint for *aps1*∆ mutants) revealed no significant differences in CFUs across the lungs, liver, kidney, spleen, and brain between mice infected with WT and *aps1*∆ strains, indicating the presence of underlying colonization in the mice (Fig. 2C). Furthermore, histological analysis of infected lung tissue revealed a similar abundance of fungal cells in mice infected with the WT, *aps1*∆ mutants, or complemented strains (Fig. 2D). Lung sections from mice infected with WT and complemented strains exhibited disruption of normal lung architecture, characterized by extensive immune cell infiltration and mucus-filled airways. In contrast, lungs from *aps1*∆ mice contained distinct pockets of yeast cells encased by densely organized immune infiltrates, resembling granulomatous containment (Fig. 2D). Quantitation of the diameter of the granulomatous regions and the average number of these regions per lung section revealed greater accumulation for mice infected with the *aps1*∆ mutant relative to mice inoculated with the WT strain (Fig. S2). Overall, these findings demonstrate that mutants lacking the small AP-1 subunit exhibited different behavior *in vivo* compared with mutants lacking the medium or large subunits, suggesting that this subunit plays distinct roles in survival and virulence in the host.

### Loss of AP-1 complex adaptins impairs the ability of *C. neoformans* to establish infection in mice

**Fig 3:**
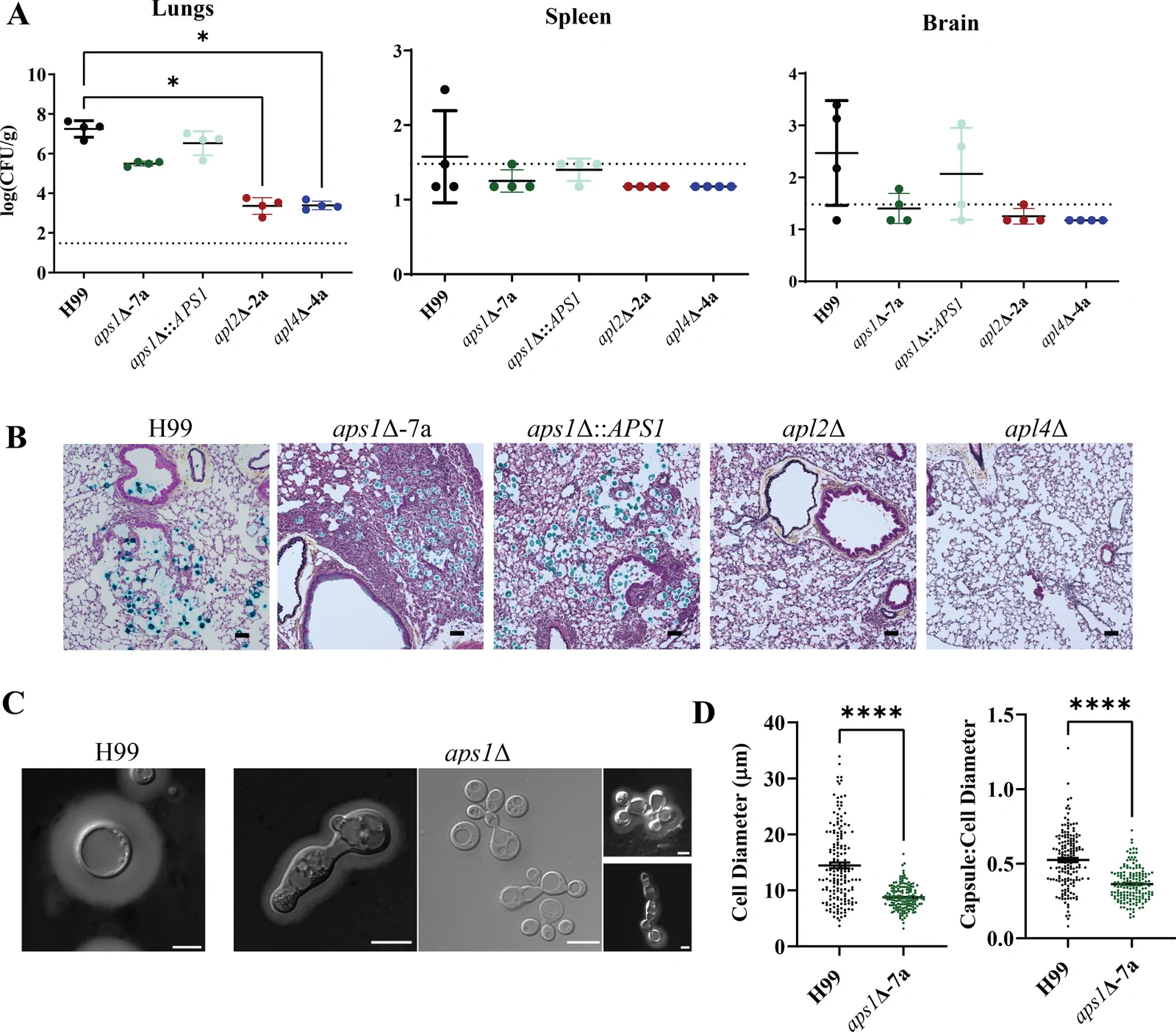
Mutants lacking AP-1 complex subunits display altered virulence during early infection, and loss of small subunit Aps1 results in abnormal cellular morphologies *in vivo*. (**A**) CFUs were measured to determine fungal burden in lungs, spleen, and brain for mice infected with WT, *aps1*Δ, *aps1*Δ::*APS1*, *apl2*Δ, or *apl4*∆, or treated with physiological saline (uninfected) at 7 dpi. The dotted black horizontal line indicates the limit of detection for each organ. Data are presented as mean ± SD. Significance indicated as *, *P* < 0.05, one-way ANOVA. (**B**) Representative histopathological micrographs of lung tissue from mice infected with WT, *aps1*Δ, *aps1*Δ::*APS1*, *apl2*Δ, or *apl4*∆ strains, collected at 7 dpi and stained with Movat’s Pentachrome. Scale bars = 100 µm. (**C**) Yeast cells of the WT strain or *aps1*Δ mutants were isolated from the lungs of infected mice and stained with India ink to visualize polysaccharide capsules. Scale bars = 10 μm. (**D**) Cell size was measured manually, and at least 100 cells were analyzed per strain. Representative differential interference contrast (DIC) micrographs are shown. Significance indicated as ****, *P* < 0.0001; one-way ANOVA. Data are representative of two independent experiments.

As described above, infections with *apl2*∆ and *apl4*∆ mutants were cleared as evidenced by undetectable CFUs in the liver, spleen, kidneys, and brain (Fig. 1C), whereas *aps1*∆-infected mice exhibited containment and long-term maintenance of colonization without overt symptoms. Given these striking differences in disease severity between mice infected with mutants lacking the large or small subunits, we next sought to understand the underlying basis for these distinct outcomes. In particular, we wondered whether large subunit mutants fail to establish infection early in the host, and whether early events specific to *aps1∆* infection might facilitate its persistence and survival *in vivo*. To address these questions, we monitored disease and measured fungal load at the early time point of 7 days post-infection (dpi). We found that the mice with the *apl2*∆ and *apl4*∆ mutants showed significantly reduced fungal loads in the lungs compared with those infected with the WT strain (Fig. 3A). In both the spleen and brain, these mutants exhibited reduced fungal burden to levels similar to those observed in the WT and the *aps1*Δ mutant. Surprisingly, mice infected with the *aps1*∆ mutant showed lung fungal burdens comparable to those of the WT and complemented *aps1*∆::*APS1* strains (Fig. 3A). Further histopathological analysis at day 7 revealed distinct lung pathologies among mice infected with the different strains (Fig. 3B). In mice infected with the WT and complemented strains, fungal cells, visualized as dark bluish green by Movat’s Pentachrome staining, were widespread around the bronchioles. By contrast, no yeast cells were detected in the lungs of mice infected with the *apl2*∆ or *apl4*∆ mutants. The lungs of *aps1*∆-infected mice displayed a distinct pathology in that pockets of yeast cells were encased by densely organized immune infiltrates at day 7, and we observed an overall disrupted lung architecture. In contrast, WT cells were largely confined to alveolar spaces, and the lungs of the infected mice showed minimal immune infiltrates. The areas of early containment for the *aps1*∆ mutant resembled the organization observed at day 40 (Fig. 2D), though less severe in terms of immune infiltration and yeast proliferation. We noted that some regions of the lung remained structurally normal and free of fungal cells at day 7 (Fig. 3B). Taken together, these results indicate that loss of the AP-1 adaptin large subunits either prevents *C. neoformans* from establishing infection or leads to rapid clearance by the host, whereas loss of the small subunit has a less pronounced effect on early infection compared with WT. These findings also highlight a critical role for AP-1 adaptins in the early stages of *C. neoformans* virulence and establishment of infection.

In infections caused by *C. neoformans*, effective macrophage-mediated control relies on early phagocytosis, which can be negatively impacted by the polysaccharide capsule and cell size (26). Morphological transitions are a common theme among pathogenic fungi, enabling them to dramatically and dynamically alter their shape and size during infection (27). Given that host immune cells encounter pathogens with highly variable and often irregular morphologies, cell size and shape become critical determinants of macrophage uptake (27). For example, there is an optimal particle size of approximately 2–3 μm for efficient phagocytosis by macrophages (28). The observation that fungal loads were similar between WT and *aps1*∆-infected mice early in infection led us to investigate morphological features of *aps1*∆ cells, including their *in vivo* cell and capsule size. Compared with WT cells isolated from infected lungs, *aps1*∆ cells exhibited significantly reduced cell body and capsule diameters (Fig. 3C and D). However, despite their small size, *aps1*∆ cells frequently exhibited abnormal elongated morphologies or formed chains and clusters within the lung (Fig. 3C). Such clusters or multicellular aggregates may overwhelm macrophages and instead favor extracellular proliferation of these cells. The *aps1*∆ mutant may also be defective in titan cell formation, and such a defect would potentially contribute to the observed altered disease progression. However, a test of the AP-1 mutants for titan cell formation in culture revealed no statistically significant defects relative to the WT strain in terms of mean cell size (Fig. S3). The percentages of cells >10 μm appeared to be lower for the mutants, although the differences were not statistically significant in comparison with the WT strain.

### AP-1 complex mutants elicit an altered immune response during early phases of infection

**Fig 4:**
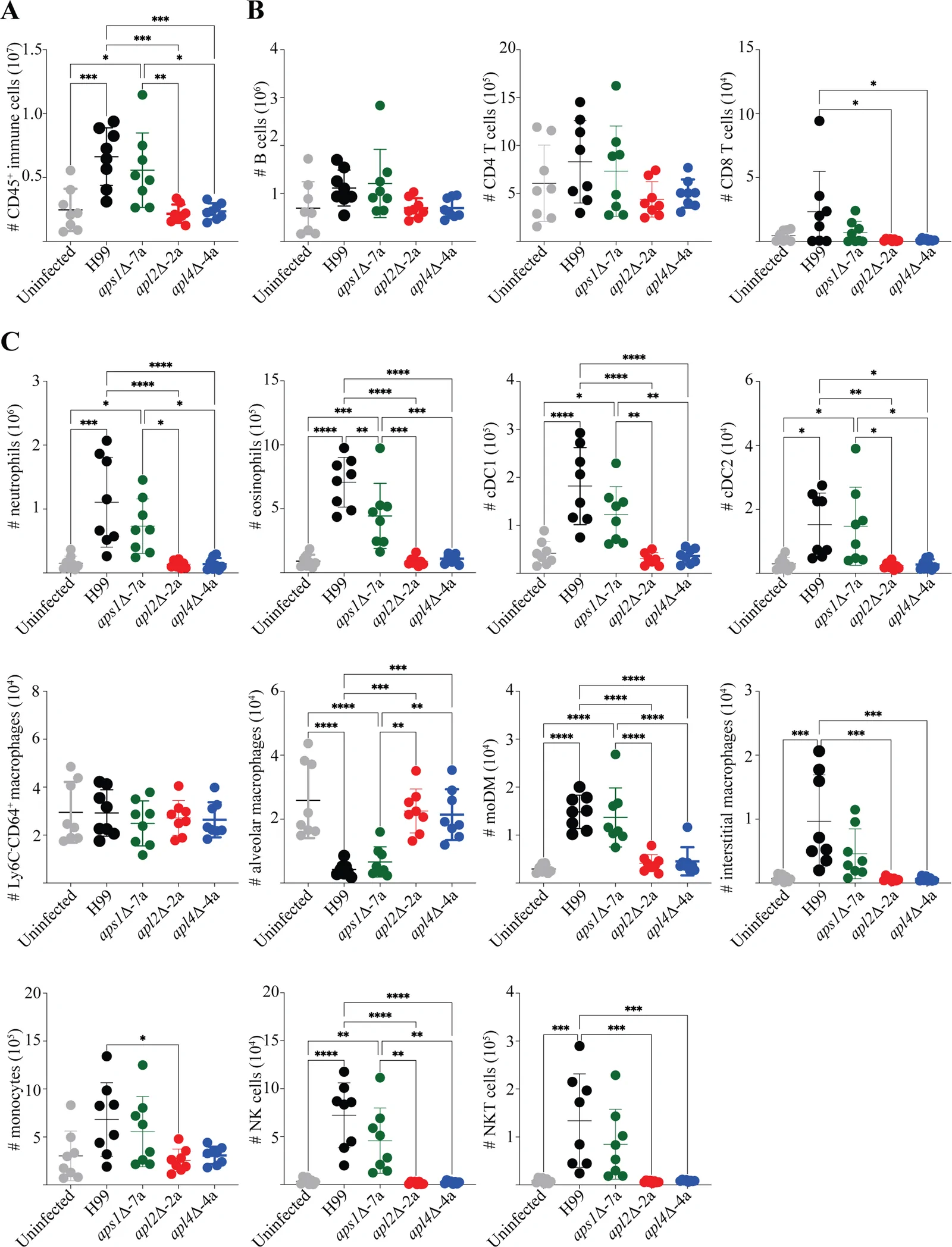
The AP-1 mutants provoke an altered immune response. Immune cell analysis in the lung tissue of BALB/c mice infected with WT, *aps1*Δ, *apl2*Δ, or *apl4*∆, or treated with physiological saline (uninfected) at 7 dpi. (**A**) Total number of CD45+ leukocytes, (**B**) adaptive CD4, CD8 T cells, and B cells, and (**C**) innate immune cells, including neutrophils, eosinophils, dendritic cells (DCs), natural killer cells (NK cells), macrophages, and monocytes in the lungs. Pooled data are presented as mean ± SD and are representative of at least two independent experiments for each time point (*n* = 4 mice/time point). Significance indicated as *, *P* < 0.05; **, *P* < 0.01; ***, *P* < 0.001; ****, *P* < 0.0001; one-way ANOVA.

To further investigate whether loss of the large AP-1 subunits impairs the ability of *C. neoformans* to establish infection or leads to rapid clearance by the immune system, and to further explore the unusual virulence pattern of the *aps1*∆ mutant, we focused our analysis on host immune responses at day 7 post-infection, a timepoint more reflective of early infection dynamics (Fig. 4). We also examined responses at day 40 (Fig. S4). Mice infected with the *apl2*∆ or *apl4*∆ mutants displayed immune profiles similar to those observed in naïve, uninfected mice at both day 7 and day 40 post-infection (Fig. 4; Fig. S4). Across nearly all immune cell populations examined, these mice exhibited markedly reduced numbers of immune cells compared with mice infected with the WT strain, except for B cells and CD4 T cells, which showed no differences between the groups (Fig. 4). Notably, alveolar macrophage numbers in mice infected with the *apl2*∆ or *apl4*∆ mutants were comparable to those in naïve mice. This pattern of alveolar macrophage responses implies that the large subunit adaptins of the AP-1 complex are required for *C. neoformans* to successfully establish infection and evade early immune clearance.

In *aps1*∆-infected mice, total CD45+ cell numbers were comparable to those of mice infected with the WT strain (Fig. 4A). T cell and B cell populations were also similar between the two groups (Fig. 4B). No significant differences were observed in the numbers of neutrophils, type 1 and type 2 conventional dendritic cells (cDC1, cDC2), monocyte-derived macrophages (moDMs), monocytes, natural killer (NK) cells, or natural killer T (NKT) cells (Fig. 4C). Notably, eosinophils were the only immune cell population that differed, and these cells were significantly reduced in *aps1*∆-infected mice compared with WT-infected mice (Fig. 4C). We further assessed disease progression by analyzing lung cytokine profiles and observed significantly reduced IL-6 and IFN-γ levels in mice infected with the *apl4*∆ or *apl2*∆ mutants, along with reduced IL-4 levels in *apl4*∆-infected mice (Fig. S5). These cytokine patterns mirror those of naïve, uninfected mice, suggesting that mutants lacking AP-1 large subunits may fail to persist long enough in the host to elicit a robust immune response.

### Loss of the AP-1 small subunit results in attenuated virulence, a pulmonary granulomatous response, and a diminished immune response at endpoint

**Fig 5:**
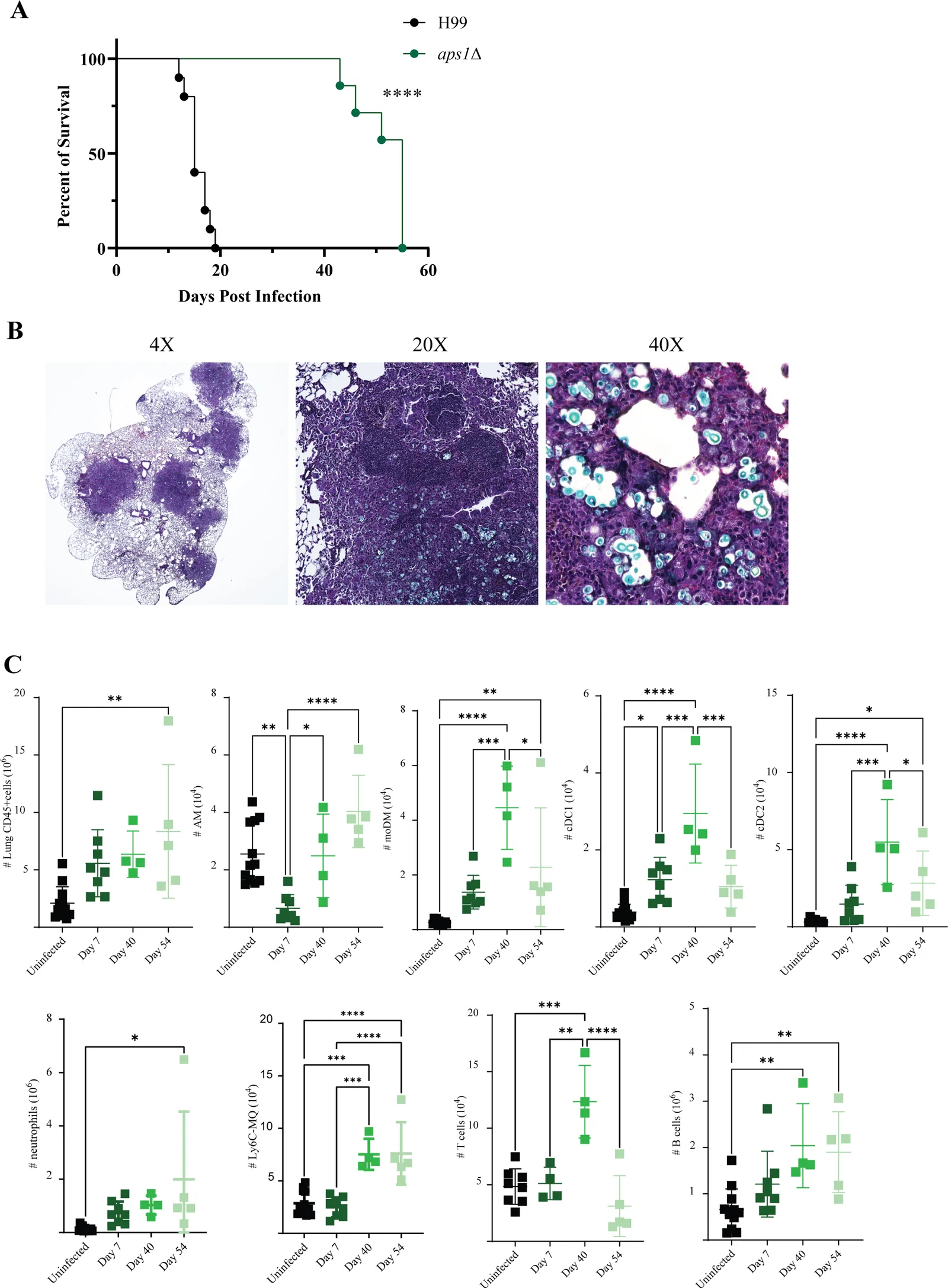
Loss of Aps1 results in attenuated virulence and a pulmonary granulomatous response. (**A**) Survival of BALB/c mice intranasally inoculated with cells of the WT (H99), or *aps1*∆ mutant strains. (**B**) Representative histopathological micrographs of lung tissue from mice infected with *aps1*∆ mutant, collected at experimental endpoint and stained with Movat’s Pentachrome. (**C**) Immune cell analysis in the lung tissue of BALB/c mice infected with *aps1*∆ or treated with physiological saline at days 7, 40, and the experimental endpoint. Total number of CD45+ leukocytes, innate immune cells including alveolar macrophages (AMs), monocyte-derived macrophages (moDMs), conventional dendritic cells (cDC1, cDC2), neutrophils, Ly6C- macrophages, and adaptive immune cells, including T cells and B cells, are shown. Data are representative of two independent experiments and presented as mean ± SD. Significance indicated as *, *P* < 0.05; **, *P* < 0.01; ***, *P* < 0.001; ****, *P* < 0.0001; one-way ANOVA or log-rank test.

**Fig 6:**
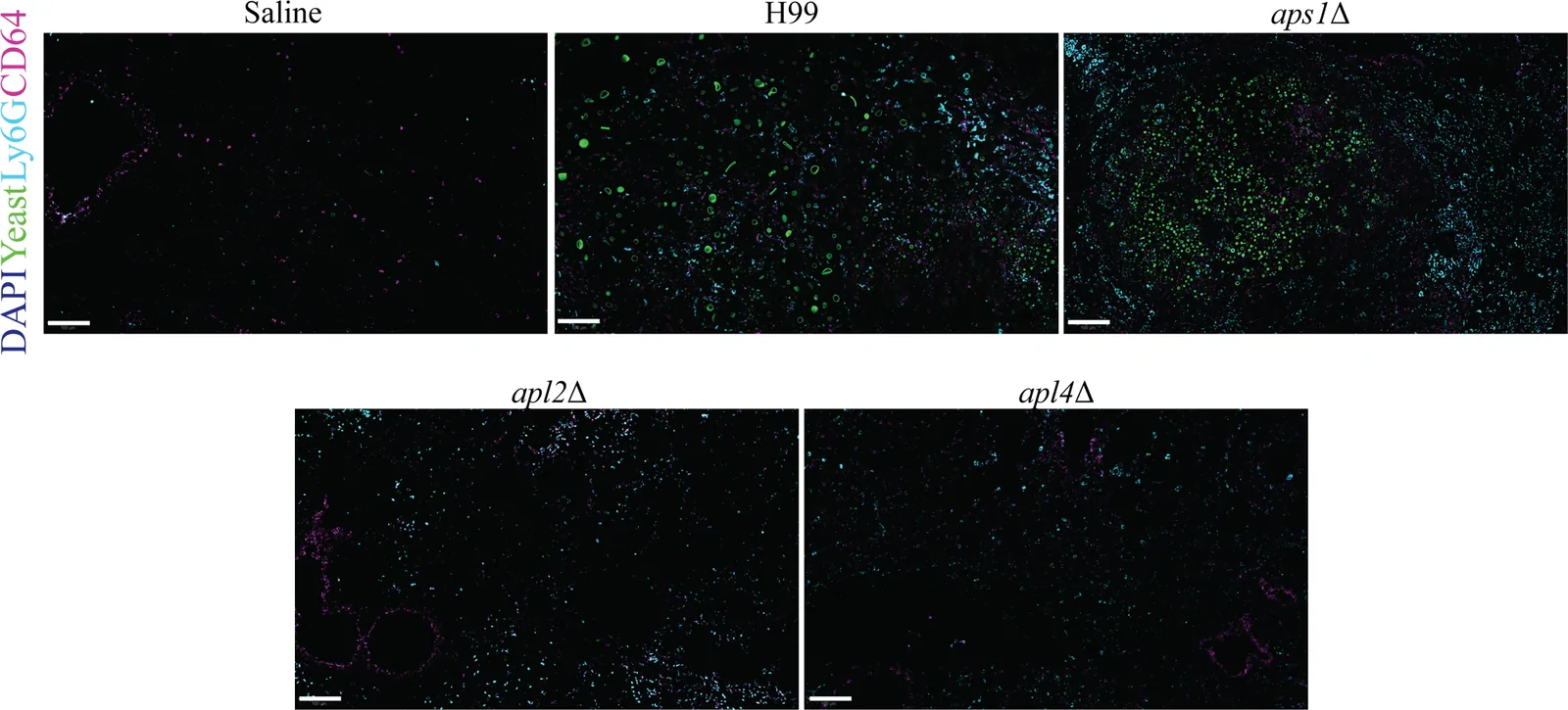
Mice infected with *aps1*∆ form granuloma-like structures composed of yeast cells surrounded by neutrophils and macrophages. Lungs from BALB/c mice infected with WT, *aps1*Δ, *apl2*Δ, or *apl4*∆ strains, or treated with physiological saline (uninfected), collected at experimental endpoints (for WT and *aps1*∆) or at day 40 (for *apl2*∆ and *apl4*∆). Sections were stained with fluorescent markers for Ly6G (Cyan, neutrophils), CD64 (magenta, macrophages), *C. neoformans* (green, SF), and nuclei (blue, DAPI). Representative images were acquired at 20× magnification. Scale bar = 100 µm.

Given that *aps1*∆-infected mice showed no overt symptoms yet had fungal burdens comparable to WT-infected mice at day 40, the typical endpoint in our experiments, we extended the infection to further examine disease progression and outcomes for mice inoculated with this mutant. We hypothesized that one of three scenarios might occur: the host would clear the fungus, the infection would remain contained by organized immune infiltrates, or the mutant would eventually overwhelm the host and cause mortality. Surprisingly, mice infected with the *aps1*∆ mutant suddenly began showing symptoms, including weight loss and increased respiratory rate at days 42 to 43, and the mice succumbed to the disease. While the WT strain caused all infected mice to succumb by day 19, mice infected with the *aps1*∆ mutant exhibited delayed disease onset and mortality, with the last mouse euthanized at day 54 (Fig. 5A). For the infection with the *aps1*∆ mutant, histology revealed patches of lung tissue with dense immune infiltrates, within which yeast cells were contained (Fig. 5B). These regions also had macrophages with elongated nuclei and multinucleated cells, key features of a granuloma (Fig. 5B). Notably, the *aps1*∆ yeast cells displayed abnormal morphologies similar to those shown in Fig. 3C. Interestingly, other areas of the lung appeared completely normal, with open alveolar spaces and no immune infiltration. Immunofluorescence staining revealed distinct differences between the lungs from WT and *aps1*∆ infections. In lungs infected with WT, Ly6G^+^ neutrophils and CD64^+^ macrophages, as well as yeast cells, were distributed throughout the tissue (Fig. 6). In contrast, lungs from *aps1*∆-infected mice exhibited a granuloma-like arrangement, with solophenyl flavine (SF)-stained yeast cells forming a central core surrounded by a defined layer of Ly6G^+^ neutrophils and CD64^+^ macrophages (Fig. 6). These data indicate that loss of the AP-1 small subunit triggers a granulomatous response that contains the infection.

We also examined immune responses in the lung at the experimental endpoint (day 54) to better understand why these mice ultimately succumbed to the infection. Flow cytometry analyses revealed that mice infected with the *aps1*∆ mutant displayed significantly different immune cell profiles at endpoint compared with days 7 and 40. At endpoint, the *aps1*∆-infected mice showed elevated numbers of CD45+ T cells, monocyte-derived macrophages (moDMs), type 2 cDC2, and B cells relative to uninfected controls (Fig. 5C). When compared with early infection (day 7), mice at the endpoint exhibited largely similar immune cell profiles except for elevated numbers of alveolar macrophages. However, *aps1*∆-infected mice at the endpoint showed reduced numbers across most immune cell subsets overall relative to both day 7 and day 40, with the exception of alveolar macrophages (Fig. 5C). Notably, moDMs, and conventional dendritic cells were decreased at endpoint compared with day 40, and total T cell numbers were also significantly diminished (Fig. 5C). These findings suggest that the immune response wanes late in infection, potentially due to sequestration of *aps1*∆ cells within granulomas.

**Fig 7:**
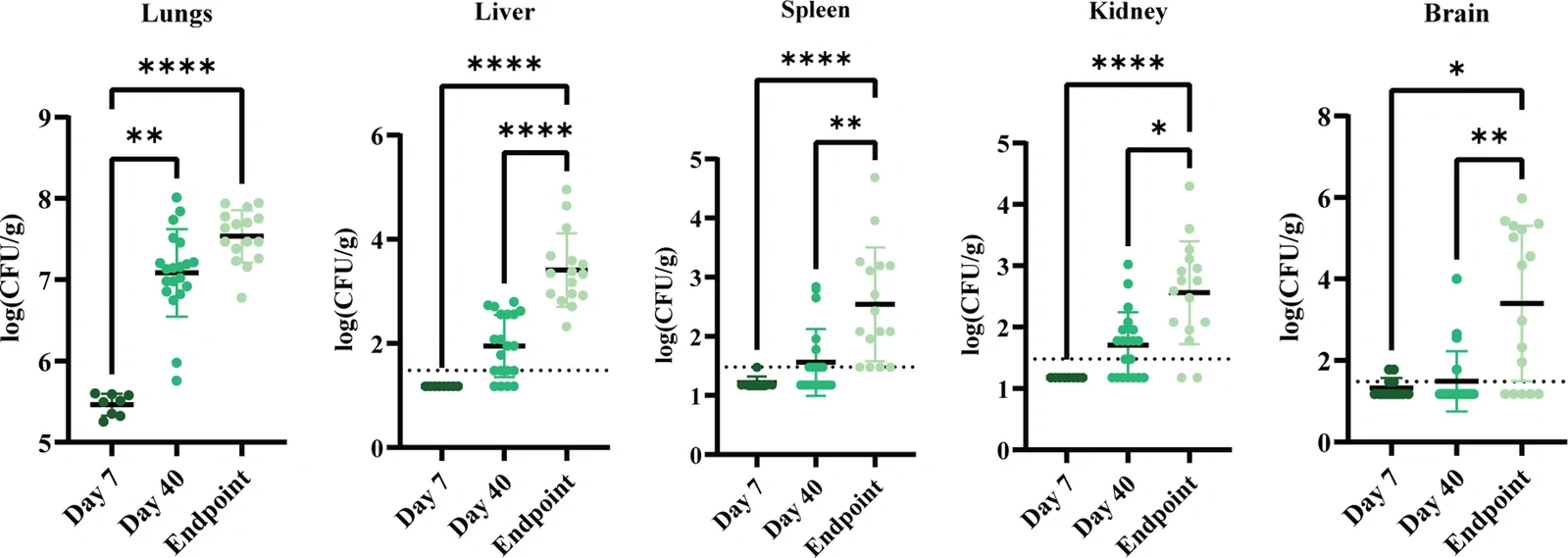
Mice infected with the *aps1*∆ mutant exhibit increased extrapulmonary dissemination at endpoint. Colony-forming units (CFUs) were measured to determine fungal burden in lungs, liver, kidney, spleen, and brain for mice infected the *aps1*∆ at days 7, 40, and at the experimental endpoint (EE). The dotted black horizontal line indicates the limit of detection for each organ. Data are representative of two independent experiments and presented as mean ± SD. Significance indicated as *, *P* < 0.05; **, *P* < 0.01; ****, *P* < 0.0001; one-way ANOVA.

Given the sudden appearance of symptoms and mortality in *aps1*∆-infected mice after day 40, we asked whether the yeast cells were breaching the granuloma containment and disseminating beyond the lung tissue. To explore this question, we examined fungal burden in lungs, liver, kidney, spleen, and brain at the experimental endpoint. The mice infected with the *aps1*∆ mutant exhibited significantly increased CFUs in the liver, kidney, spleen, and brain at the experimental endpoint but not in the lungs compared with day 40 (Fig. 7). Notably, fungal loads in these mice did not differ between days 7 and 40, indicating that the extrapulmonary dissemination occurs rapidly and late during infection (Fig. 7).

Taken together, these findings suggest that the *aps1*∆ mutant experiences a shift in disease progression after day 40. This shift is characterized by a diminishing immune response and abrupt systemic fungal dissemination, which likely contributes to mortality in these mice.

### Loss of the AP-1 small subunit results in lung damage in infected mice

**Fig 8:**
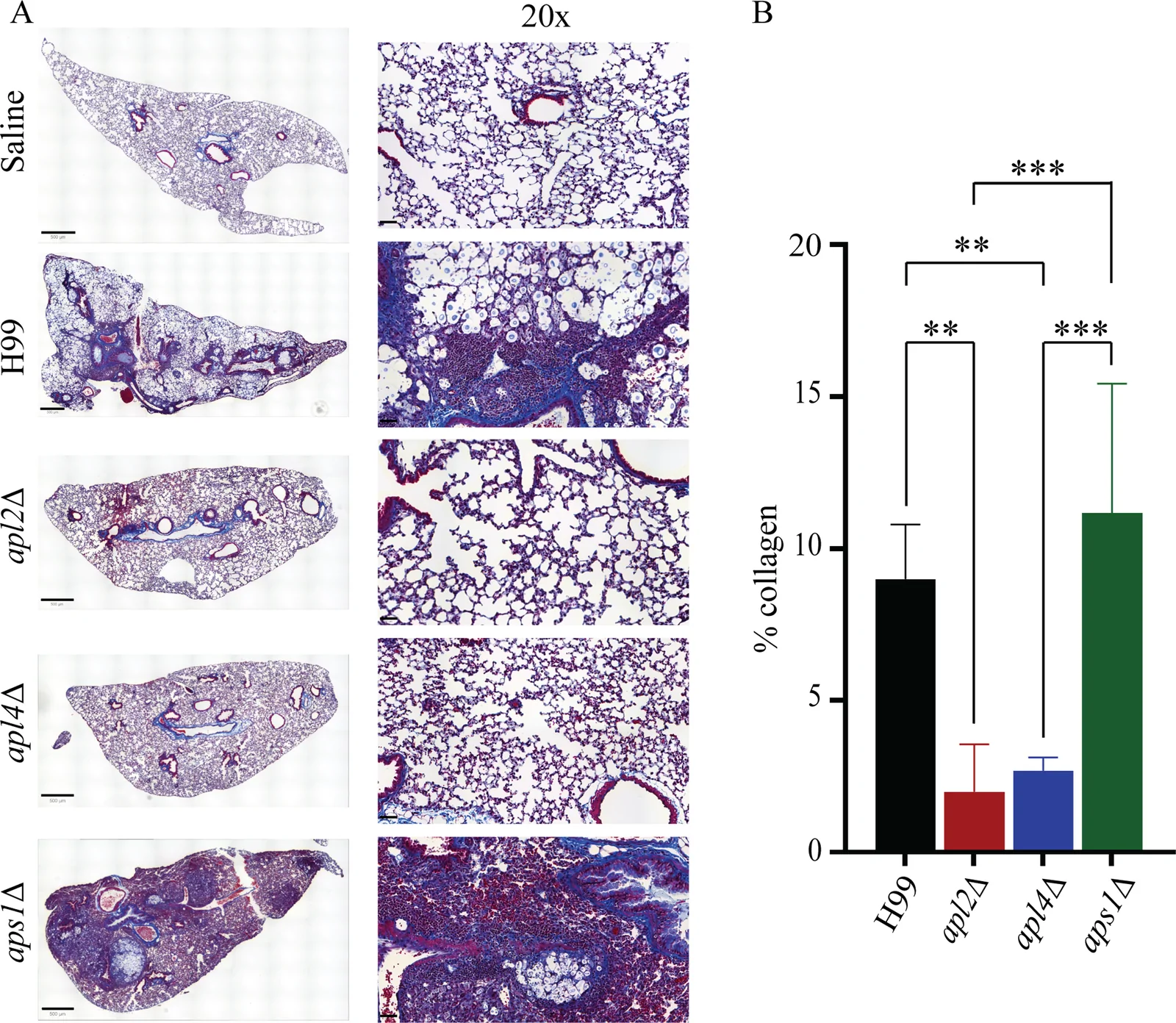
Lungs of mice infected with the *aps1*∆ mutant show increased collagen deposition. (**A**) Representative histopathological micrographs of lung tissue from mice infected with WT, *apl2*∆, *apl4*∆, or *aps1*∆ collected at experimental endpoint (EE) or day 40 (*apl2*∆ and *apl4∆*) and stained with Masson’s Trichrome to visualize collagen. Scale bar: 2× = 500 µm (left panels); 20× = 50 µm. (**B**) Quantification of collagen deposition in lungs at the experimental endpoint. Five randomly selected fixed regions per sample, excluding airways and blood vessels, were analyzed. Color segmentation was applied, and the sum of collagen-positive pixels was divided by total pixels within the analyzed regions to determine the percentage of positive staining. Data are representative of two independent experiments and presented as mean ± SD. Significance indicated as **, *P* < 0.01; ***, *P* < 0.001; one-way ANOVA.

We next sought to investigate the mechanism underlying extrapulmonary dissemination in mice infected with the *aps1*∆ mutant. *C. neoformans* has evolved various strategies to escape the lung, including exploiting macrophages and monocytes as “Trojan horses” to carry the yeast cells across the respiratory epithelium (29). Tissue damage can also enable the yeast to bypass the lung mucosa and enter the bloodstream or lymphatic system (29–31). Previous studies using human alveolar epithelial cell lines have shown that *C. neoformans* can damage lung epithelial cells (30). Given that abnormal collagen deposition is an indicator of chronic inflammation and lung injury, we hypothesized that the mice infected with the *aps1*∆ mutant experience abrupt but rapid dissemination as a result of lung tissue damage. Consistent with this idea, lungs from the infected mice displayed abnormal and significantly increased collagen deposition at both day 7 and the experimental endpoint (Fig. 8; Fig. S6).

## DISCUSSION

In this study, we used mutants lacking the AP-1 complex subunits (Apl2, Apl4, and Aps1 in particular) to demonstrate that the complex is required for virulence in a murine inhalational model of cryptococcosis. We also showed that mutants deficient in AP-1 subunits display distinct patterns of infection progression and disease outcome. Specifically, mutants lacking the large adaptin subunits Apl2 and Apl4 fail to establish infection in the host and are rapidly eliminated by the immune system. In contrast, yeast cells lacking the small subunit, Aps1, persist in the lung, likely establishing a form of latent infection, and become sequestered within granuloma-like structures. Given that our *in vitro* characterization of AP-1 complex mutants revealed different severities of phenotypes between large and small subunit mutants, we hypothesized that their contributions to virulence would likewise differ (24). Our findings in this study confirmed this prediction.

Our observation that the AP-1 complex contributes to virulence is consistent with roles attributed to the complex in other fungal and parasitic pathogens. In the phytopathogenic fungus, *Botrytis cinerea*, the AP-1 complex has been implicated in vesicular trafficking, cell wall integrity, and virulence. More specifically, *B. cinerea* mutants lacking the large subunit exhibit impaired cell wall integrity and are unable to produce the infectious structures required for successful host invasion and colonization (32). The AP-1 complex is essential in *Trypanosoma brucei* and required for virulence in *Leishmania* spp. (25, 33, 34). In *T. cruzi*, ablation of the AP-1 γ-adaptin (large subunit) leads to Golgi retention of Cruzipain, a key virulence factor in Chagas disease (25). In *Toxoplasma gondii*, disruption of the medium subunit of the AP-1 complex results in missorting of microneme proteins, which are required for host invasion and intracellular survival (35). Similarly, in *L. mexicana*, AP-1 subunits are essential for host infectivity and flagellar biogenesis, and their loss results in hypersensitivity to elevated temperature and perturbations in membrane lipid composition (21). Finally, we note that in the malaria parasite *Plasmodium falciparum*, the AP-1 complex is required for intracellular growth, formation of specialized invasion organelles, and trafficking of rhoptry proteins and other secreted factors required for host cell invasion and intracellular survival, as well as for the generation of invasive progeny (36, 37). Parasites lacking the AP-1 medium subunit also display defects in cell division in which daughter cell budding occurs normally but the cells fail to segregate completely (35). This cell division phenotype is also present in *C. neoformans* AP-1 complex mutants.

We also found that the mutant lacking the small AP-1 subunit exhibits a distinct pattern of infection progression and outcome compared with mutants lacking the large subunits. In particular, mice infected with the *aps1*∆ mutant are impaired in their ability to clear the infection and instead contain the yeast within granuloma-like structures characterized by multinucleated cells and macrophages. The yeast cells remain enclosed by these host cells. We hypothesize that the inability of these mice to eradicate *aps1*∆ cells results in long-term persistence in the lung in granuloma-like structures. Granuloma formation is thought to represent a protective host response that limits fungal access to blood vessels and prevents extrapulmonary dissemination (38). However, recent research shows that granulomas are not passive containment structures but rather dynamic sites of host-microbe interaction in which the host attempts to restrict microbial proliferation, while the pathogen exploits the granuloma as microniche for long-term survival and remains protected from immune recognition (39).

We propose that distinct features of the *aps1*∆ mutant contribute to its persistence in the lung, including a potentially reduced growth rate, altered cell surface structure that could modulate the host immune response, greater thermotolerance relative to large subunit mutants, and abnormal cell morphology in which cells cluster and/or grow in chains that may influence interactions with immune effector cells (24). Cell surface features are key determinants of how the immune system detects and responds to fungal pathogens. For example, cell wall β-glucan can bind to receptors on macrophages, neutrophils, and lymphocytes, and modulate downstream signaling, activate lymphocyte effector functions, and facilitate rapid recruitment of immune cells to the cite of infection (40). Changes in capsule polysaccharide structure or trafficking of cell surface proteins may also play a role. For example, Cas35 is found at higher concentrations on the *aps1*∆ cell surface, as evidenced by increased fluorescence intensity of mCherry-Cas35 in *aps1*∆ genetic background (24). It is also possible that the mutant exhibits altered secretion of immunomodulatory fungal proteins, such as Cpl1 (41). These ideas remain speculative and require further investigation. With regard to the underlying molecular mechanisms for distinct Aps1 functions, it is possible that the small subunit has interaction partners that mediate functions independent of the AP-1 complex. Certainly, physical interaction studies with each of the individual complex subunits summarized in the *Saccharomyces* genome database reveal shared and distinct interaction partners (42). Given different interaction partners and the less severe phenotypes observed in the *aps1*Δ mutant *in vitro*, it is also possible that the AP-1 complex retains some functionality in the absence of the small subunit (24). That is, the large and medium subunits may still assemble into a complex in the absence of Aps1, whereas loss of the large adaptin subunits would more severely disrupt complex formation and function. The individual subunits may also make different contributions to cargo selection and it is known, for example, that the medium subunit recognizes canonical tyrosine-based (YXXφ) sorting motifs, while the hemicomplex consisting of the large and small subunits recognizes dileucine-based ([D/E]XXXL[L/I/M]) motifs (43).

The observation that the loss of large adaptin subunits, but not the small subunit, impairs the ability of *C. neoformans* to establish disease in mice is consistent with different severities of phenotypes between large and small subunit mutants observed in our *in vitro* characterization experiments (24). Our studies demonstrated that stress response and virulence factor production differ among the individual AP-1 subunit mutants. While *apl2*∆ and *apl4*∆ display pronounced sensitivity to elevated temperature, vacuolar stress, and divalent cation stress, the *aps1*∆ mutant is comparatively more resistant and generally exhibits milder phenotypes. In particular, the *aps1*∆ mutant was impaired for growth on rich (YPD) medium or RPMI or DMEM at 37°C relative to the WT strain, but the *apl2*∆ and *apl4*∆ mutants showed much greater proliferation defects (24). These differences may partly explain the different virulence outcomes observed in mice.

In this study, we further demonstrated that mice infected with the *aps1*∆ show elevated extrapulmonary dissemination which is associated with a diminished immune response at later stages of infection. The mechanisms underlying fungal reactivation from granulomatous containment remain poorly understood (39). The *C. neoformans gcs1*∆ mutant, which lacks glucosylceramide synthase, is known to induce pulmonary granulomas in mice that can later reactivate and disseminate following immunosuppression (38). Similarly, the *mar1*∆ mutant, which lacks the *MAR1* gene required for cell surface remodeling during infection, also induces a pulmonary granulomatous response leading to chronic, persistent infection (39). Similar to *aps1*∆ mutants in this study, the *mar1*∆ mutant displays altered cell surface architecture and elicits altered immune responses. Notably, *mar1*∆ provokes WT-like pulmonary cytokine responses and immune cell profiles early during infection, which is also consistent with the behavior of our *aps1*∆ mutant (39). Other *C. neoformans* mutants with prolonged disease progression have been reported (44–46). For example, mice infected with mutants lacking the transcription factor Usv101 exhibited extended survival and eventual death from pneumonia rather than meningitis (44). Mutants lacking Usv101 exhibit a number of virulence-related phenotypes, including altered capsule, melanin, and titan cell formation (44, 45). Additionally, the *PDR6* gene encodes an atypical pleiotropic drug resistance transporter and mutants lacking the gene provoke an altered disease profile upon infection of mice (46). As with the *aps1*∆ mutant, mice infected with the *pdr6*∆ mutant survive longer compared with infection with the wild-type strain, and the mutant cells were contained in granuloma-like structures later in the infection. Mice eventually succumbed to disease caused by the mutant due to a hyper-inflammatory immune response leading to pneumonia, a finding that may be explained in part by cell surface changes for the mutant. We note that comparisons between the different *C. neoformans* mutants may be complicated by the use of different mouse strains in the various studies.

In humans, cryptococcal infection is often subclinical, and low levels of yeast cells can persist in the murine lung following primary infection (47). The term “cryptococcal granuloma,” has been historically used to describe any mass lesion associated with *C. neoformans* infection. These structures tend to be quite heterogeneous and difficult to classify, and they can include necrotic or fibrotic granulomas (47). While we did not observe necrotic lesions in the structures containing the *aps1*∆ mutant cells, further characterization is needed. Granulomatous responses are known to be essential for controlling infections by *Cryptococcus* and many pathogens (47). However, early granulomatous responses are also known to promote bacterial growth in infections caused by *Mycobacterium tuberculosis,* for example (48). Additional evidence of proliferation within granulomas comes from an *in vitro* granuloma model demonstrating that *Candida albicans* is not cleared by immunocompetent host immune cells but instead proliferates and persists within a highly complex immune environment (49). One possibility is that *aps1*∆ cells persist in the lung and later reactivate once the immune response declines, as seen in the late stages of infection. What remains unclear is the mechanism underlying the tipping point that disrupts the balance between immune-mediated containment and eventual proliferation and dissemination of the *aps1*∆ mutant.

Finally, we showed that infection with the *aps1*∆ mutant results in extensive lung damage, as evidenced by enhanced collagen deposition in the lung. Excessive or prolonged pro-inflammatory response can cause tissue injury (50). Because cytokine levels and pulmonary immune cell profiles do not differ significantly between mice infected with the *aps1*∆ and WT strains, and because *aps1*∆ cells persist significantly longer in the lung, we speculate that the persistent exposure to inflammatory cytokines and immune cell effector activity over the extended course of infection contributes to the observed histopathology. Importantly, structural tissue damage to the lung can compromise tissue integrity and give yeast cells increased access to the blood vessels, facilitating dissemination beyond the lung. This would in part explain the extrapulmonary dissemination observed in *aps1*∆-infected mice during later stages of infection.

Although we have clarified the distinct contributions of the large and small AP-1 subunits to fungal virulence, the role of the medium subunit remains unknown. Future studies should explore how this subunit contributes to virulence. We recently presented evidence that AP-1 complex mediates trafficking of proteins involved at the cell surface (24). Given the unique immune responses observed at late infection stages, it is possible that AP-1 complex is involved in the secretion of proteins with immunomodulatory properties. This would be consistent with observations in parasites, where this complex is involved in trafficking of rhoptry proteins and other secreted effectors that are immunogenic and important players in host-pathogen interactions (36, 51–53). Identifying the cargo proteins transported by this complex will be an important first step toward understanding the mechanisms underlying the virulence and immune modulation exhibited by the *aps1*∆ mutant. It is also possible that the AP-1 complex influences the trafficking of factors that influence capsule structure. Overall, our findings demonstrate that the AP-1 complex deficiencies negatively impact *C. neoformans* virulence, reveal distinct contributions of individual subunits, and identify this complex as a potential target for therapeutic intervention in cryptococcosis.

## MATERIALS AND METHODS

### Mice

Female BALB/c mice (4–6 weeks old) were obtained from Charles River Laboratories (Pointe-Claire, Quebec, Canada) and maintained in the Modified Barrier Facility (MBF) at the University of British Columbia (UBC). Mice were housed in sterilized cages with microisolator tops under specific pathogen-free conditions, with unrestricted access to food and water.

### Murine infection and assessment of virulence

Fungal cells were cultured overnight in 5 mL of YPD medium at 30°C, washed three times with physiological saline, and resuspended in saline (pH 7.4). A suspension containing 2 × 10^5^ cells in 50 μL was administered intranasally to each mouse. Following inoculation, mice were monitored daily. Animals that reached the experimental endpoint, defined as a 15% reduction in body weight, were euthanized with an overdose of isoflurane followed by carbon dioxide asphyxiation. Differences in virulence were evaluated using long-rank survival analysis. For fungal burden determination, the lungs, liver, spleen, kidneys, and brain were collected, weighted, and homogenized in two volumes of 1× PBS using a MixerMill (Retsch, Cole-Parmer, Montreal, Canada). Serial dilutions of tissue homogenates were plated on YPD agar supplemented with 50 μg/mL chloramphenicol, and colony-forming units (CFUs) were quantified after 48 h of incubation at 30°C.

### Histology and immunofluorescence (IF)

Lung tissues were collected and fixed overnight in 10% buffered formalin. Fixed samples were dehydrated, embedded in paraffin, sectioned, and stained with hematoxylin and eosin (H&E), Movat’s Pentachrome, or Masson’s Trichrome. Images were acquired using a Nikon ECLIPSE Ti2 inverted microscope equipped with a DS-Ri2 high-resolution full-frame sensor camera (Nikon Instruments Inc.) or Leica Thunder 3D Imager. Image visualization and processing were performed with the NIS-Elements Viewer or QuPath software. Sections stained with Movat’s Pentachrome were analyzed for quantification of granulomatous region diameter. Granulomatous regions were identified by the presence of yeast cells enclosed within an outer boundary of multinucleated giant cells. For each lung section, the number of granulomatous regions were also determined. Quantification was performed using ImageJ FIJI software (54) on lung sections from at least five mice per strain at each time point.

For assessment of lung damage and quantification of collagen content, Masson’s Trichrome-stained sections were used. Five randomly selected fixed regions, excluding large conducting airways and blood vessels, were analyzed per sample. Color segmentation was applied, and the sum of all collagen-positive pixels was divided by the total number of pixels within the analyzed areas to determine the percentage of positive staining.

For immunofluorescence, sections were deparaffinized and incubated at 95°C for 20 min in citrate buffer for antigen retrieval. Slides were then washed with PBS, blocked with 10% goat serum (Sigma), and stained with antibodies against CD64 (1:200, clone 1; ThermoFisher Scientific) and Ly6G (1:100; clone 1A8-Ly6G; ThermoFisher Scientific), followed by goat anti-rabbit AlexaFluor 647 (1:200; ThermoFisher Scientific) and goat anti-rat AlexaFluor 568 (1:200; ThermoFisher Scientific). Nuclei were stained with DAPI, and yeast cell walls were stained using solophenyl flavine 7GFE 500 (SF). Images were acquired using a Leica Thunder 3D imager and analyzed with the open-source software QuPath.

### Cytometric bead array (CBA) assay

Lungs were excised and collected in 1x PBS supplemented with 2x cOmplete, EDTA-free protease inhibitor cocktail (Roche). Tissues were homogenized using a mixer mill, and supernatants of homogenates were collected for cytokine analysis using the BD Cytometric Bead Array (CBA) Mouse Th1/Th2/Th17 kit (BD Biosciences). The kit was used for the simultaneous detection of mouse interleukin-2 (IL-2), interleukin-4 (IL-4), interleukin-6 (IL-6), interferon-γ (IFN-γ), tumor necrosis factor (TNF), interleukin-17A (IL-17A), and interleukin-10 (IL-10) in a single sample. All procedures were carried out according to the manufacturer’s instructions. Briefly, beads coated with seven cytokine-specific capture antibodies were first prepared. For each reaction, 50 μL of the mixed capture beads, 50 μL of lung supernatant or cytokine standard, and 50 μL of phycoerythrin (PE) detection reagent were added to assay tubes and incubated for 2 h at room temperature in the dark. Samples were then washed with 0.5 mL of wash buffer, centrifuged, and the resulting bead pellet was resuspended in 300 μL of assay buffer. Fluorescence was measured on a CytoFLEX S flow cytometer (Beckman Coulter) equipped with four lasers (405, 488, 561, and 633 nm) and FITC (525/40), PE (585/42), and ECD (600/20) filters. Cytokine concentrations were determined from fluorescence intensities using calibration curves generated from serially diluted standards.

### Lung processing and immune infiltration assays

Lungs were perfused with 15–20 mL of ice-cold 1x PBS (pH 7.4), excised, minced, and enzymatically digested in 5 mL of RPMI 1640 containing 0.7 mg/mL collagenase IV and 50 μg/mL DNase I (Worthington) for 1 hour at 37°C. The digested tissue was passed through a 70-μm cell strainer. Red blood cells were lysed using RBC lysis buffer (10 mM Tris, 0.84% NH_4_Cl) for 5 min at room temperature. The homogenate was filtered through a 35-μm strainer to obtain a single-cell suspension. Cells were blocked with 2.4G2 supernatant to inhibit nonspecific Fc receptor binding and stained for 20 min at 4°C with fluorescently labeled antibodies targeting surface antigens in flow cytometry buffer (PBS with 2% BSA and 2 mM EDTA). Multiparametric flow cytometry was performed using an Attune NxT Flow Cytometer (Thermo Fisher Scientific) as described previously (55), and data were analyzed with FlowJo software.

The following fluorophore-conjugated antibodies against mouse antigens were used: CD45 (30-F11), CD24 (M1/69), CD11c (N418), CD11b (M1/70), CD64 (X54-5/7.1), Ly6C (HK1.4), Ly6G (1A8), CD4 (RM4-5) from BioLegend; NK1.1 (PK136), SiglecF (1RNM44N), MHCII (M5/114.15.2) from eBioscience; CD3 (145-2C11), CD8 (53-6.7), and CD19 (1D3) from AbLab (UBC). Viable cells were identified using the Zombie Aqua fixable viability kit (BioLegend), and absolute cell counts were determined using 123count eBeads (eBioscience/Thermo Fisher Scientific). Statistical analysis was performed using GraphPad Prism 10.0, with one-way ANOVA used for comparison of biological replicates.

### Induction of titan cell formation

Strains were cultured overnight in 5 mL of Sabouraud dextrose broth (SB) (Becton, Dickinson and Company, Franklin Lakes, NJ, USA) at 30°C and 150 rpm. Cells were then washed twice with MOPS (50 mM) and resuspended to 1 × 10⁴ cells/mL in 1 mL of modified Titan Cell Medium (TCM) (56), consisting of 5% SB, 5% heat-inactivated FBS, adjusted to pH 7.3 with MOPS (50 mM). Cells were incubated at 37°C in 5% CO_2_ for 16–18 h. Subsequently, cells were stained with India ink and imaged with a Zeiss Plan-Apochromat 63× or 100×/1.46 oil objectives on a Zeiss Axioplan 2 microscope, coupled with a CMOS camera (ORCA-Flash4.0 LT; Hamamatsu Photonics). Cells sizes were measured using Fiji (ImageJ 1.53q).

### Statistical analysis

Statistical analyses were performed using Kruskal-Wallis and Mann-Whitney tests, log-rank tests, and one-way ANOVA. All analyses were conducted in GraphPad Prism.

## SUPPLEMENTAL MATERIAL

Supplemental figures iai.00100-26-s0001.pdf Fig. S1 to S6.

ASM does not own the copyrights to Supplemental Material that may be linked to, or accessed through, an article. The authors have granted ASM a non-exclusive, world-wide license to publish the Supplemental Material files. Please contact the corresponding author directly for reuse.
