# Supplementary material for: Subunits of adaptor protein complex-1 make distinct contributions to the virulence of Cryptococcus neoformans
Source: Infect Immun. 2026 Aug 4;94(9):e00100-26. doi: 10.1128/iai.00100-26 (PMC13552105; doi:10.1128/iai.00100-26)
Supplement: Supplementary file 1 [file iai.00100-26-s0001.pdf]

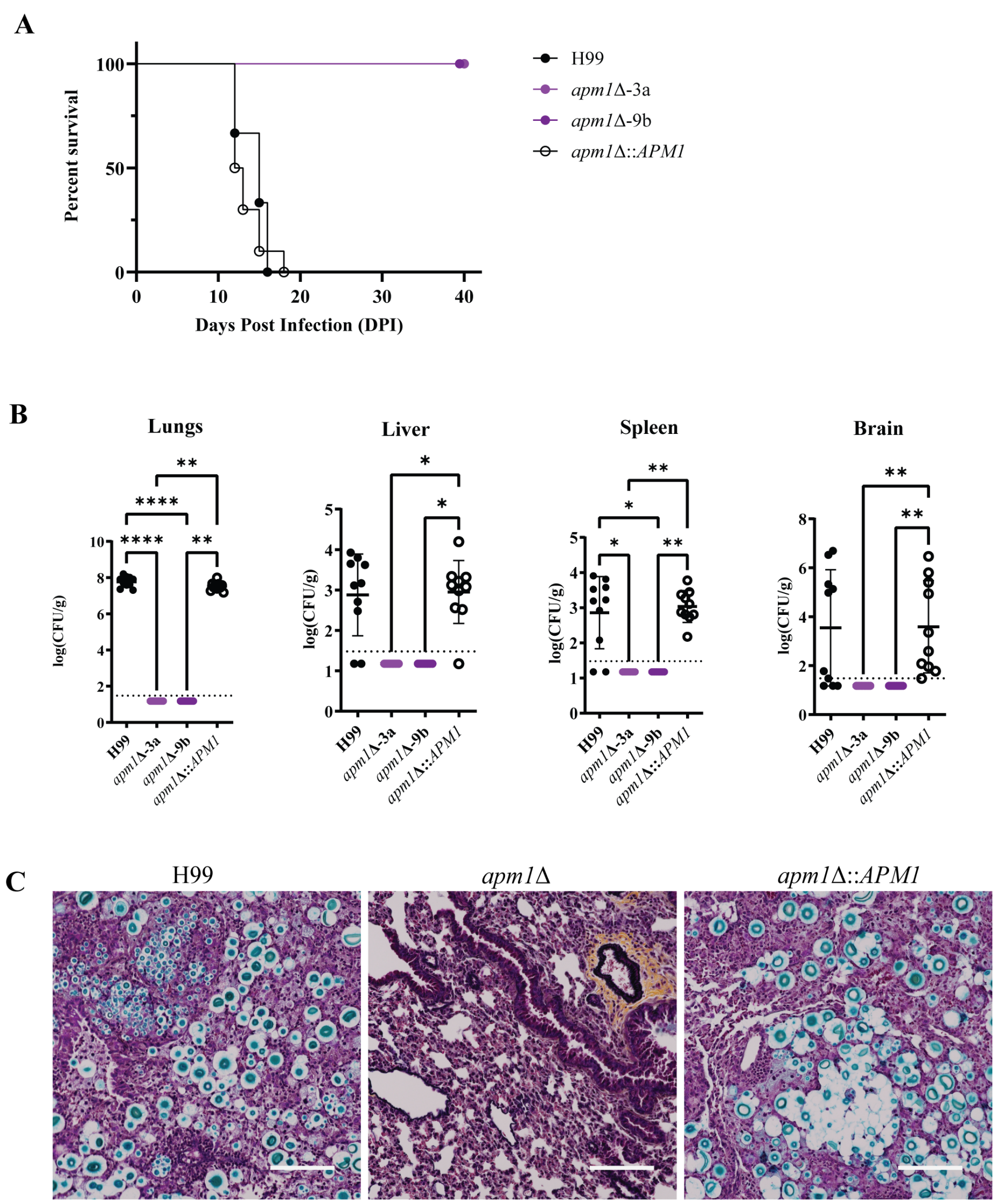

Figure S1. Mutants lacking the medium subunit of the AP-1 complex are avirulent. (**A**) Survival of BALB/C mice intranasally inoculated with cells of the WT (H99), *apm1*Δ (two independent mutants), or the *apm1*Δ::*APM1* (complemented) strains (B) The fungal burden for mice infected with each strain were determined by measuring colony forming units (CFUs). The dotted black line indicates the limit of detection for each organ. (**C**) Representative histopathological micrographs of lung tissue from mice infected with WT, *apm1*Δ, or *apm1*Δ::*APM1* complemented strains, collected at experimental endpoint and stained with Movat's Pentachrome. Scale bars = 100 µm. Data are presented as mean ± SD. Significance indicated as *, $P < 0.05$; **, $P < 0.01$; ****, $P < 0.0001$; one-way ANOVA.

A

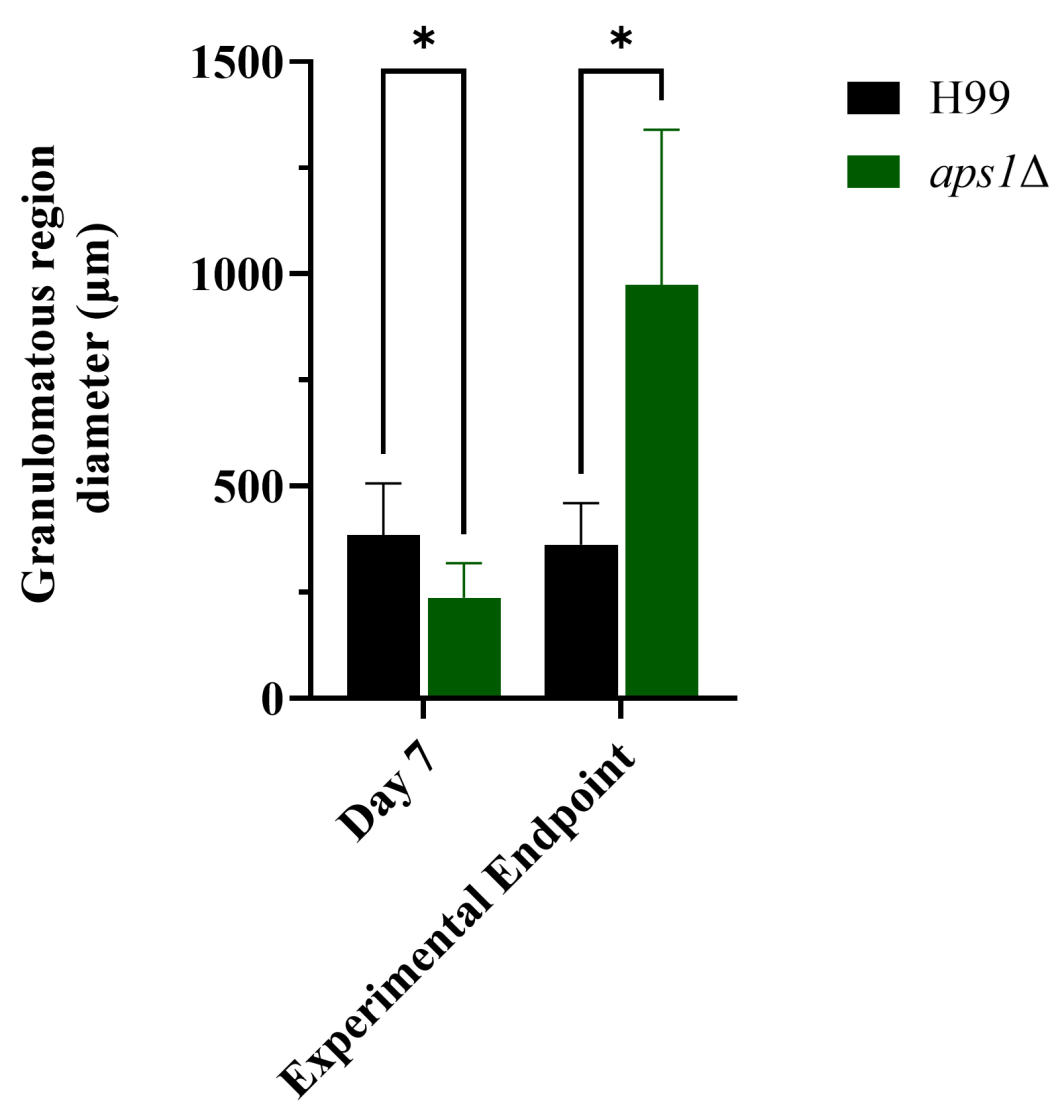

B

Mean granulomatous region diameter (µm)

Average number of granulomatous regions/section

	7 DPI	40 DPI	Endpoint
**H99**	385.3<br>3.5		361.6<br>3.2
***aps1*Δ**	236.8<br>5	456.9<br>7.7	973.9<br>4.2

Figure S2. Loss of Aps1 results in a pulmonary granulomatous response. (**A**) Quantification of granulomatous region diameter of BALB/c mice intranasally inoculated with cells of WT (H99), or *aps1*Δ mutant strains at the indicated timepoints. Data are representative of lung sections from at least five mice per strain per time point and presented as mean ± SD. Significance indicated as *, P < 0.05; unpaired Student *t-test* (**B**) Summary of the mean granulomatous region diameter and the average number of granulomatous regions per lung section of BALB/c mice inoculated with the indicated strains at days 7 and 40 (DPI, days post inoculation), and at the experimental endpoint (EE).

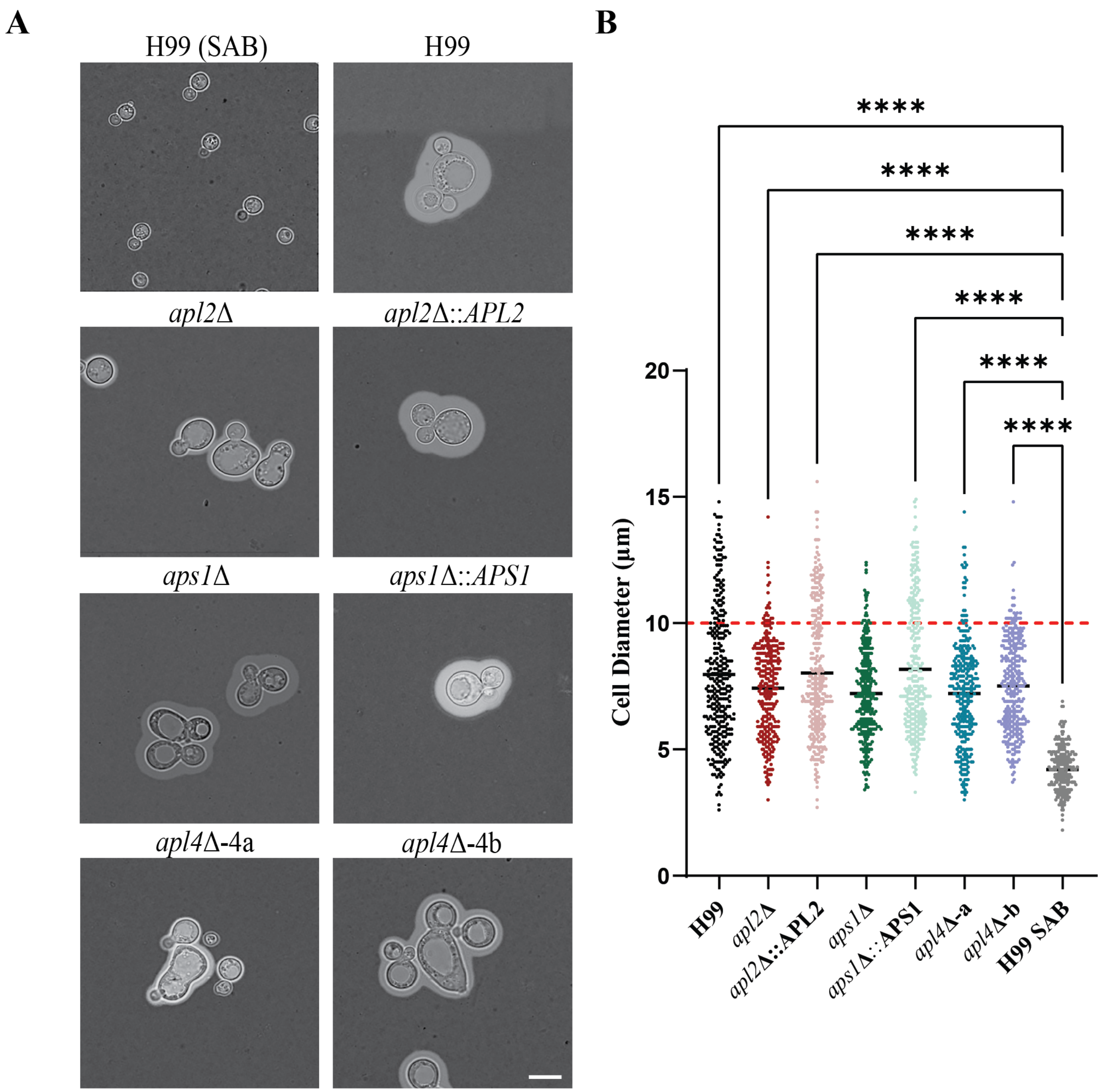

Figure S3. The AP-1 complex is not required for titan cell formation. (A) Indicated strains were grown overnight in Sabouraud dextrose broth (SAB) at 30 °C with shaking at 200 rpm. Cultures were inoculated at 1×10^4 cells/mL into modified Titan Cell Medium (TCM) or SAB and incubated at 37 °C in 5% CO_2 for 16–18 h. Representative India ink-stained micrographs from at least three independent experiments are shown (n ≥100 cells examined per experiment). Scale bar, 10 µm. (B) Quantification of cell diameters for *C. neoformans* strains following titan cell induction in TCM (as in panel A). Data represent three independent experiments. Statistical significance was assessed by Kruskal–Wallis test with Dunn's multiple comparisons test (****, $P < 0.0001$).

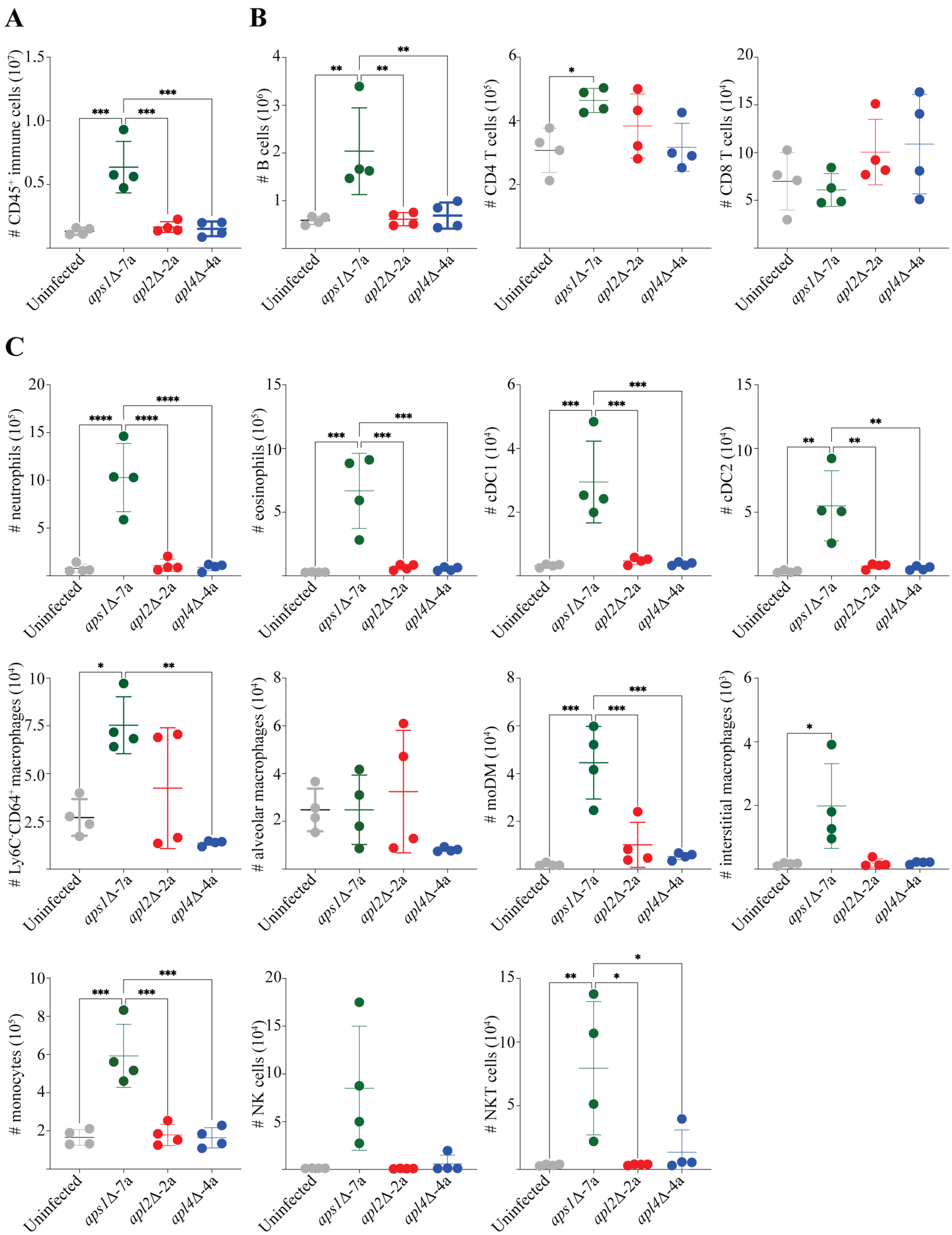

Figure S4. The AP-1 mutants provoke an altered immune response. Immune cell analysis in the lung tissue of BALB/c mice infected with *aps1*Δ, *apl2*Δ, or *apl4*Δ, or treated with physiological saline (uninfected) at 40 dpi. (**A**) Total number of CD45+ leukocytes, (**B**) adaptive CD4, CD8 T cells, and B cells and (**C**) innate immune cells, including neutrophils, eosinophils, moDMs and monocytes in the lungs. Data are presented as mean ± SD (n = 4 mice/time point). Significance indicated as *, $P < 0.05$; **, $P < 0.01$; ***, $P < 0.001$; ****, $P < 0.0001$; one-way ANOVA.

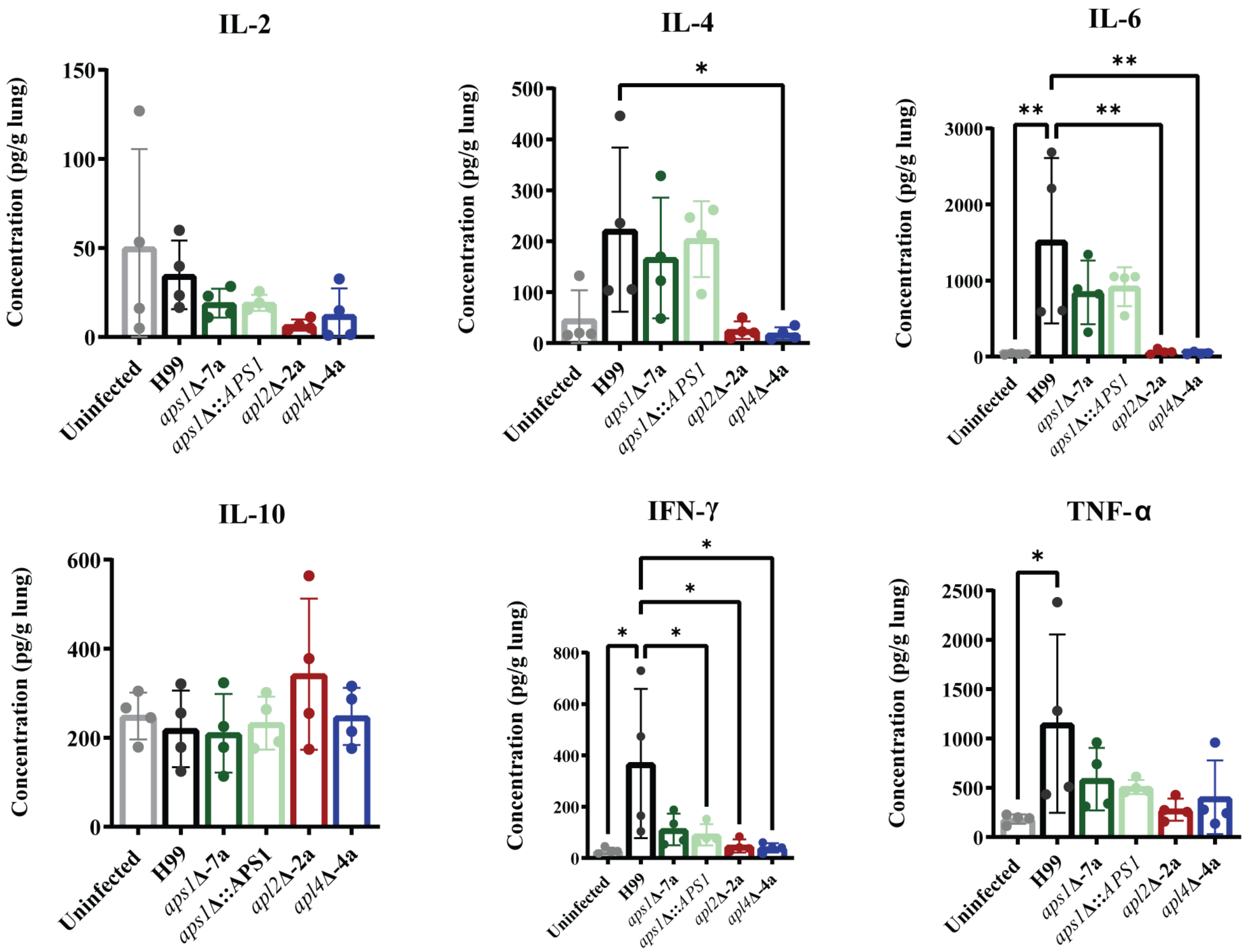

Figure S5. Loss of adaptin subunits results in altered cytokine profiles in infected mice. Lung cytokine profiles of BALB/c mice infected with indicated strains or treated with physiological saline at 7 days post infection. Cytokines were extracted from lung tissue using a mixer mill and quantified using a cytometric bead array (CBA) mouse inflammation kit. Data are presented as mean ± SD, (n = 4) and are representative of two independent experiments. Significance indicated as *, $P < 0.05$; **, $P < 0.01$; one-way ANOVA with Tukey's *post hoc* test.

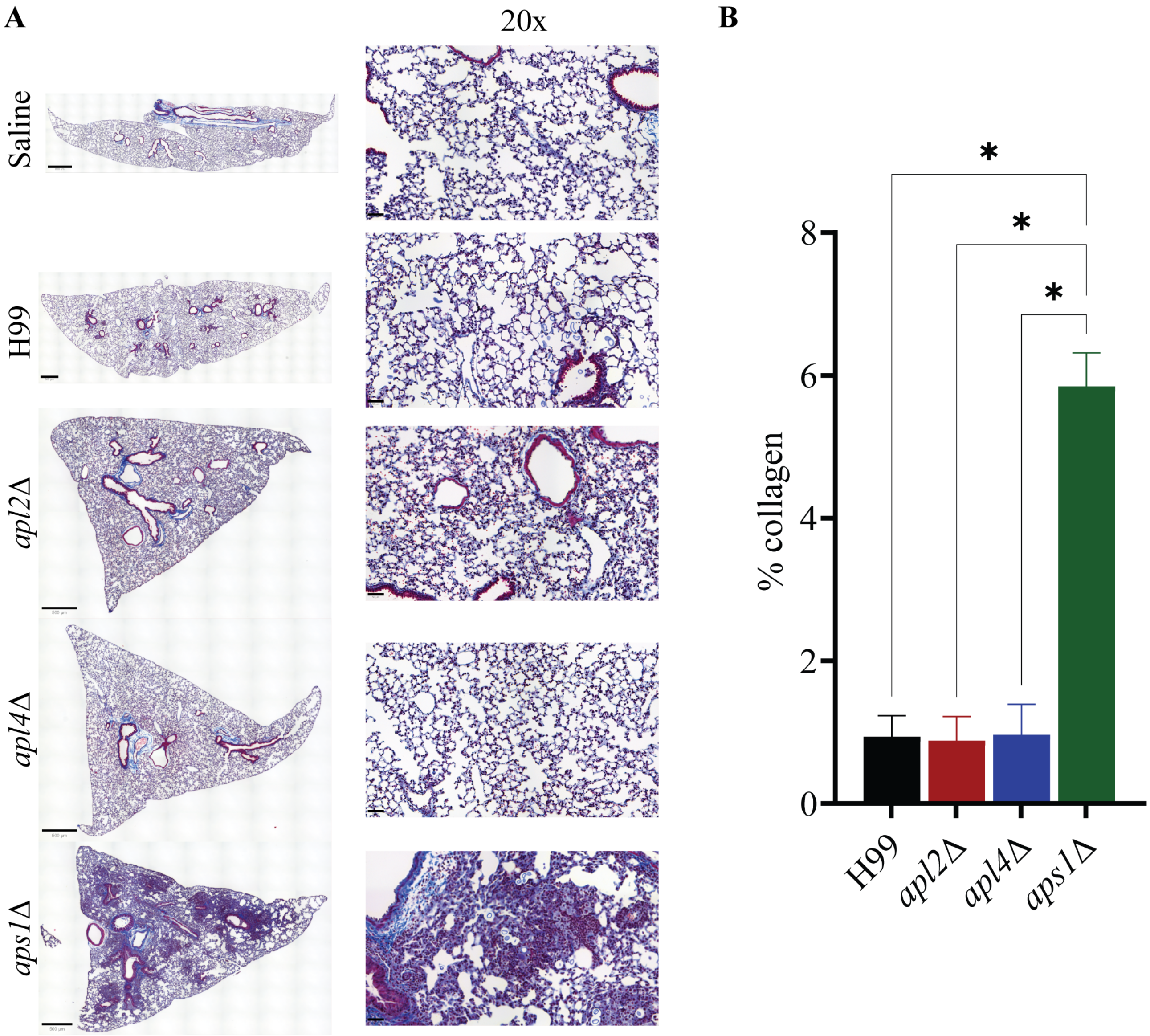

Figure S6. Lungs of mice infected with *aps1*Δ show increased collagen deposition. (**A**) Representative histopathological micrographs of lung tissue from mice infected with WT, *apl2*Δ, *apl4*Δ, or *aps1*Δ collected at day 7 post-infection and stained with Masson's Trichrome to visualize collagen. Scale bar: 2X = 500 µm; 20X = 50 µm. (**B**) Quantification of collagen deposition in lungs at day 7. Five randomly selected fixed regions per sample, excluding airways and blood vessels, were analyzed. Color segmentation was applied, and the sum of collagen-positive pixels was divided by total pixels within the analyzed regions to determine the percentage of positive staining. Data are representative of two independent experiments and presented as mean ± SD. Significance indicated as *, $P < 0.05$; one-way ANOVA.
